# Improvement of a real-time RT-PCR assay for the detection of enterovirus RNA

**DOI:** 10.1186/1743-422X-6-95

**Published:** 2009-07-07

**Authors:** Marian AC Piqueur, Walter A Verstrepen, Peggy Bruynseels, An H Mertens

**Affiliations:** 1 Department of Microbiology, ZNA Hospitals, site Middelheim, Lindendreef 1, 2020 Antwerp, Belgium

## Abstract

We describe an improvement of an earlier reported real-time RT-PCR assay for the detection of enterovirus RNA, based on the 5' exonuclease digestion of a dual-labeled fluorogenic probe by Taq DNA polymerase. A different extraction method, real-time RT-PCR instrument and primer set were evaluated. Our data show that the optimized assay yields a higher sensitivity and reproducibility and resulted in a significant reduced hands-on time per sample.

## Findings

Enteroviruses are responsible for a substantial number of aseptic meningitis and encephalitis, especially in neonates and infants [1,2]. In recent years, a number of rapid diagnostic tests for enterovirus have been developed, including different RT-PCR assays [3,4].

Our study group earlier reported a real-time RT-PCR assay for the detection of enterovirus RNA, based on the 5' exonuclease digestion of a dual-labeled fluorogenic probe by Taq DNA polymerase. This assay has now been further improved by comparing different extraction methods and real-time RT-PCR instruments. We also evaluated a different primer set to reduce the number of possible mismatches in the highly conserved region of the 5' UTR from the enterovirus genome [5,6].

Primers (Gibco-BRL, Merelbeke, Belgium) and probes (Eurogentec, Seraing, Belgium) were designed using Primer Express 1.5 software and were directed to the conserved sequences in the 5'UTR of the enterovirus genome. The 5' and 3' end of the probe was labelled with the reporter 6-carboxyfluorescein (FAM) and quencher 6-carboxytetramethylrodamine (TAMRA) respectively (see table 1).

**Table 1:** Compared primer-probe sets

	Primer-probe set 1 (earlier reported)	Primer-probe set 2
Forward primer	5'-CCCTGAATGCGGCTAATCC-3' Genbank: NC_002058.3 (452–470)	5' CCGGCCCCTGAATGC-3' Genbank: NC_002058.3 (447–461)

Reverse primer	5'-ATTGTCACCATAAGCAGCCA-3' Genbank: NC_002058.3 (596–577)	5' CACCGGATGGCCAATCCA-3' Genbank: NC_002058.3 (639–622)

Probe	FAM 5' AACCGACTACTTTGGGTGTCCGTGTTTC-3' TAMRA Genbank: NC_002058.3 (535–562)	

The OptiQual Enterovirus Run Control (Acrometrix Europe, Alkmaar, The Netherlands) enabled us to monitor the whole procedure from extraction to final analysis. Enterovirus RNA was extracted both manually using QiaAmp viral RNA kit (Qiagen, Hilden, Germany) as reported previously and automated on NucliSens EasyMAG extractor (bioMérieux, Marcy L'Etoile, France), according to the instructions of the manufacturer. Real-time RT-PCR was performed on the ABI Prism 7700 SDS using the TaqMan One-step RT-PCR kit (Applied Biosystems, Foster City, California) versus the LightCycler 480 instrument using the TaqMan RNA Amplification kit (Roche Diagnostics, Mannheim, Germany). A log 0.4 serial dilution of the Enterovirus Control was prepared in OptiQual Diluent resulting in concentrations of 100.0, 39.8, 15.8, 6.3, 2.5 and 1.0 Enterovirus Units (EVU) per ml. From each concentration 8 replicates were extracted with both extraction methods and each RNA extract was analysed on both real-time PCR instruments. Additionally, primer-probe set 1 and primer-probe set 2 were compared in each setting.

When the undiluted OptiQual Enterovirus Run Control (100 EVU/ml) was tested on the LightCycler 480 instrument (maximum of 2nd derivative method) it consistently yielded lower Ct/Cp-values as compared to the ABI Prism 7700 SDS (see table 2). Extraction with Nuclisens EasyMag extractor yielded lower Ct/Cp values and less variation than the manual extraction with QiaAmp viral RNA kit but only when tested on the LightCycler 480. In our hands primer-probe set 2 was associated with a better precision in all settings.

**Table 2:** Mean Ct/Cp values and %CV values for the undiluted OptiQual Enterovirus Run Control in different real-time RT-PCR settings

Extraction Method	Platform	Mean Ct/Cp (%CV)	
		
		Primer-probe set 1	Primer-probe set 2
QiaAmp	7700 SDS	38.47 ± 3.94 (10.2%)	37.74 ± 1.35 (3.6%)
	
	LC 480	33.69 ± 2.95 (8.8%)	34.48 ± 1.67 (4.8%)

EasyMag	7700 SDS	38.23 ± 3.88 (10.2%)	36.29 ± 0.91 (2.5%)
	
	LC 480	32.81 ± 0.25 (0.8%)	32.78 ± 0.20 (0.6%)

The limit of detection (LOD) was defined as the lowest concentration (EVU/ml) still detectable with 95% confidence (probit analysis; SPSS 15.0, Chicago, Illinois). The combination of EasyMag NucliSens extractor and primer-probe set 2 on LightCycler 480 showed the lowest LOD of 4.5 EVU/ml (see table 3).

**Table 3:** Limit of detection (EVU/ml) for different RT-PCR settings obtained with OptiQual Enterovirus Run Control

Extraction Method	Platform	LOD (EVU/ml)	
		
		primer-probe set 1	primer-probe set 2
QiaAmp	7700 SDS	119.5	111.8
	
	LC 480	54.2	11.1

EasyMag	7700 SDS	43.4	17.5
	
	LC 480	13.5	4.5

To confirm our findings an enterovirus control of a different serotype, Echovirus 5 RNA Control (Vircell, Santa Fe Granada, Spain), was used to determine the dynamic range and within-run reproducibility of two real-time RT-PCR assays with NucliSens EasyMAG Extractor, Light Cycler 480 and a different primer-probe set 1 or 2. Eight replicates of a dilution series log 10^-2^, 10^-3^, 10^-4^, 10^-5^, 10^-6^, 10^-7^, 10^-8 ^diluted in nuclease free water were tested 8 fold.

In a real-time RT-PCR assay with primer-probe set 1, the lowest detectable dilution of 10^-2 ^corresponded to a mean threshold cycle of 20.32, while the highest detectable dilution factor of 10^-6 ^corresponded to a Ct/Cp-value of 34.87. Using primer-probe set 2, we obtained a mean Ct/Cp-value of 21.32 for the dilution of 10^-2 ^and a Ct/Cp-value of 36.52 for a dilution factor 10^-6 ^(fit point method). Linear regression plot showed a strong linear correlation between the obtained Ct/Cp-values and the log_10 _of the undiluted control (R^2 ^= 0.984 and R^2 ^= 0.924 for primer-probe set 1 and 2 respectively). The dynamic range of the assay spanned at least 5 logs for both primer-probe sets.

Within-run reproducibility, obtained by analyzing 10 replicates of a 10^-4 ^dilution of Echovirus 5 RNA Control was 1.64% and 0.82% for primer-probe set 1 and 2 respectively.

To further compare both primer-probe sets on various enterovirus serotypes, the Quality Control for Molecular Diagnostics (QCMD) 2008 Enterovirus and Parechovirus RNA External Quality Assessment (EQA) proficiency panel containing blanc samples, Echovirus 16, Coxsackievirus A16, A24 and B3, Poliovirus type 3 and Enterovirus 71 was analysed with both primer-probe sets using NucliSens EasyMAG extractor and LightCycler 480. All tested enterovirus serotypes were adequately detected by both primer-probe sets except for Coxsackievirus A24, which was not detected by primer-probe set 2. Parechovirus RNA is not recognised by our designed primer-probe sets [7].

In conclusion, our data obtained using OptiQual Enterovirus Run Control revealed substantial differences in sensitivity and reproducibility between different compared RT-PCR settings. Therefore the control can be used to optimize a complete real-time RT-PCR procedure from extraction to analysis during method development and subsequently as daily run control.

RNA extraction with NucliSens EasyMAG extractor and real-time RT-PCR analysis on LightCycler 480 showed a higher sensitivity and precision and yielded a lower limit of detection as compared to our earlier reported assay. In addition, introducing an automated extraction procedure resulted in significant reduced hands-on time per sample.

Although results from OptiQual Enterovirus Run Control suggest a higher sensitivity of primer-probe set 2, we were not able to confirm this with Echovirus 5 RNA Control. We maintain primer-probe set 1 in our optimized assay but in our opinion analysis with primer-probe set 2 can be useful in the case of equivocal results. Further investigation however should be performed on the detection of Coxsackievirus A24.

## Competing interests

The authors declare that they have no competing interests.

## Authors' contributions

MP carried out the laboratory experiments, interpreted the results and wrote the manuscript. WV defined the study, designed the primers, analyzed the data, co-interpreted the results and co-wrote the manuscript. PB and AH contributed their ideas to the design of the study and the manuscript. All authors read and approved the final manuscript.
